# Use of a New Ocular Insert versus Conventional Mydriasis in Cataract Surgery

**DOI:** 10.1155/2013/849349

**Published:** 2013-05-30

**Authors:** C. Torrón, P. Calvo, O. Ruiz-Moreno, J. Leciñena, A. Pérez-Iñigo

**Affiliations:** Department of Ophthalmology, Miguel Servet University Hospital, Aragonés Institute of Health Sciences, University of Zaragoza, Isabel la Católica 1-3, 50009 Zaragoza, Spain

## Abstract

*Background*. To compare the efficacy and safety of a new ocular insert versus conventional mydriasis in cataract surgery. *Methods*. We selected 70 patients undergoing cataract surgery. Thirty five patients (Group 1) received instillation of mydriatic drops (tropicamide 1%, phenylephrine 10%, and cyclopentolate 1%) prior to surgery, and 35 patients (Group 2) had a Mydriasert insert (Théa Pharma) (0.28 mg of tropicamide and 5.4 mg of phenylephrine hydrochloride) placed in the inferior fornix. Pupil size before and after surgery, blood pressure, and heart rate were measured. *Results*. Before surgery, pupil diameter was 9.44 ± 1.17 mm in Group 1 and 9.05 ± 1.54 in Group 2 (*P* > 0.05). Twenty four hours after surgery, pupil diameter was 5.20 ± 1.54 mm in Group 1 and 3.33 ± 1.15 in Group 2 (*P* < 0.001). There were no statistically significant differences in blood pressure or heart rate between groups. *Conclusions*. The effect of the Mydriasert insert was similar to conventional mydriatic agents. Pupil size was restored to normal faster when using the Mydriasert insert compared with conventional mydriatic agents for pupil dilation.

## 1. Introduction

Pharmacologic mydriasis can be induced by an adrenergic stimulant such as phenylephrine, which acts on the dilator musculature of the iris, or by an acetylcholine antagonist such as tropicamide, which allows for relaxation of the sphincter. These topical agents, however, cause nonocular reactions: for example, dry mouth, tachycardia, headache, and allergic reactions have been reported with the use of tropicamide. Phenylephrine is an alpha-receptor adrenergic agonist used locally for ocular disorders because of its vasoconstrictor and mydriatic actions. Cardiovascular reactions occur with phenylephrine, primarily in elderly patients, and include a marked increase in blood pressure, syncope, myocardial infarction, tachycardia, and arrhythmia [1, 2].

Mydriasert (Théa Pharma), developed to induce preoperative mydriasis, is an ophthalmic insert that contains 0.28 mg of tropicamide and 5.4 mg of phenylephrine hydrochloride. It is an insoluble minimatrix coated with a semipermeable membrane that increases local tolerance and regulates the release rate of the active ingredients [3].

 Mydriasert is placed in the inferior conjunctival fornix 60 minutes prior to surgery.

The purpose of this study was to evaluate the efficacy and cardiovascular safety of this new ophthalmic insert compared with the standard preoperative mydriatic agents in cataract surgery.

## 2. Methods

The Ethics Committee for Clinical Investigation of Aragon (CEICA) approved the study design. All of the participants provided written informed consent, and the study methodology followed the guidelines of the Helsinki Declaration. A total of 75 eyes of 75 consecutive subjects who were scheduled for routine cataract surgery were prospectively enrolled. We wanted to show the reality of routine cataract surgery, so we did not exclude any type of cataract. Any allergy to mydriatic agents was the only exclusion criterion. Three of the subjects did not provide informed consent, and two did not complete the tests included in the exploration protocol. Therefore, 70 eyes of white European-origin subjects were included in the statistical analysis. When both eyes fulfilled the inclusion criteria, only one eye per subject was randomly selected. Patients were randomized into two groups. Group 1 received the standard practice of instillation of mydriatic drops: a combination of tropicamide 1%, phenylephrine 10%, and cyclopentolate 1% was instilled at 15-minute intervals for 1 hour preoperatively. In Group 2, the Mydriasert insert was placed in the lower fornix 1 hour and removed before the surgery by the nurse. Three senior surgeons performed all of the procedures and were blind to the dilation treatment.

All operations were done under topical anaesthesia (tetracaine 0.1% and oxybuprocaine 0.4%), and no miotic agents were used at the surgery.

Pupil measurements were performed using the Palomar pupillometer, by a masked observer, and the light intensity was the same throughout all procedures. Blood pressure and heart rate were measured every 15 minutes 4 times after beginning the dilatation procedure, and pupil photographs were taken before and after surgery. Patient characteristics and comorbidities (arterial hypertension, diabetes mellitus, alpha-antagonist treatment, and pseudoexfoliation) were noted in the preoperative assessment. 

*Statistical Analysis. *All statistical analyses were calculated using IBM SPSS (version 19.0; IBM Corporation, Somers, NY) statistical software. All of the variables studied followed a normal distribution as verified with the Kolmogorov-Smirnov test (K-S of 1 sample). Patient characteristics were compared between groups using the Chi-square test. Pupil size, blood pressure, and heart rate were analyzed using a *t*-test.

## 3. Results

Table 1 shows the patient characteristics and comorbidities of both groups. There were no differences between groups (*P* > 0.005, Chi-square test) in age, female (%), arterial hypertension, insulin-dependent diabetes mellitus (IDDM), noninsulin-dependent diabetes mellitus (NIDDM), pseudoexfoliation, or alpha-antagonist use.

Table 2 shows the pupil diameter at each time point in both groups. Fifteen minutes after starting treatment, pupil dilation in Group 1 (drops) was 5.78 ± 1.86 and that in Group 2 was 4.64 ± 1.49 mm (*P* = 0.011, *t*-test). There were no statistically significant differences in the other measurements between groups except for pupil size 24 hours after surgery (pupil diameter 5.20 ± 1.54 mm in Group 1 and 3.33 ± 1.15 mm in Group 2 (*P* < 0.001)). 

No differences were found about the pupil size just before or after cataract surgery among the two groups. However, intraoperative data were not recorded. No other methods (pharmacological or mechanical) for pupil dilation were used during the surgeries. 

Table 3 shows the blood pressure and heart rate measurements. No significant differences were detected between groups at any time (*P* > 0.05, *t*-test).

Surgery complications were collected (posterior capsule rupture, flaccid iris, and iris herniation) and were considered homogenous between groups (*P* > 0.05, analysis of variance [ANOVA]). No patient was unable to tolerate the insert. 

## 4. Discussion

Our findings indicated that the Mydriasert insert achieves good pupil dilatation before cataract surgery, comparable to the standard set of preoperative mydriatic agents. At 15 minutes after beginning the pupil dilation treatment, pupil size was greater in patients receiving instillation of mydriatic drops (Group 1, 5.78 ± 1.86 mm) than in those treated with the Mydriasert insert (Group 2, 4.64 ± 1.49 mm). This finding was expected because the ophthalmic insert gradually releases the two active ingredients, phenylephrine and tropicamide, and at least 1 hour is needed to reach maximum pupil dilatation [4, 5].

One limitation of this study is that we used one additional topical drug in Group 1 (cyclopentolate) as long as Mydriasert only contains tropicamide and phenylephrine. We did not change our standard set of mydriatic drops in purpose to reproduce the habitual conditions of preoperative cataract patients. However, the fact that there were no statistical differences in preoperative dilatation, with or without cyclopentolate, allows us to recommend eliminating the use of this drug preoperatively.

The pupil returned to its normal size more quickly in patients using the Mydriasert insert than in those using topical drops. Installing cyclopentolate, it is known that the pupil needs 24 hours to return to normal size. Due to the long-acting effects of this drug, the pupil often remains dilated, and vision is blurred until the day after cataract surgery. The effects of the Mydriasert insert, however, disappear within a few hours.

The Mydriasert insert, with a small oblong cylinder shape, is insoluble, sterile, and biocompatible. It is inserted into the inferior conjunctival sac following instillation of one drop of anesthetic. Another advantage of the insert is that this method requires only two simple maneuvers, one to insert and one to withdraw the device, thus reducing patients' discomfort and saving time for the nursing staff, compared to having to administer drops to patients every 15 minutes. In all cases, the nurses were able to retrieve the device, and no history of ocular discomfort or ocular irritation was reported. 

The blood pressure and heart rate measurements did not differ significantly between groups. These results are consistent with those of Morgado et al. [6] and might be due to difficulties in determining the actual cause of increased blood pressure during surgery. 

Systemically administered phenylephrine, a powerful *α*-adrenergic receptor agonist, leads to peripheral vasoconstriction, resulting in increased systolic and diastolic blood pressure. Topical phenylephrine 10% increases blood pressure by up to 10 mmHg in 2% of subjects [7]. Absorption across the conjunctiva and nasal mucosa avoids first-pass metabolism in the liver and results in peak levels only 10 minutes after topical administration [8]. Nevertheless, reports of side effects in the adult population are rare in view of its widespread use.

Additional benefits may include that, during the study period, the nursing staff found application of Mydriaert a simple, efficient, and one-off event, saving time in a busy cataract unit.

In summary, the effects of the Mydriasert insert are comparable to those of standard mydriatic eyedrops in terms of cardiac safety and pupil dilation, and recovery of the normal pupil size is fast. Therefore, Mydriasert is a good alternative option for pupillary dilation prior to cataract surgery.

## Figures and Tables

**Table 1:** Clinical characteristics of both study groups.

	Group 1: Drops	Group 2: Mydriasert	*P*
Age (years)	75.53	75.44	0.969
Female	55.6%	67.9%	0.317
Arterial hypertension	50%	50%	1
IDDM	14.7%	10%	0.212
NIDDM	11.8%	14.8%	0.726
Pseudoexfoliation	2.8%	15.4%	0.072
Alpha-antagonist use	6.5%	3.8%	0.661

*N *	35	35	

IDDM: insulin-dependent diabetes mellitus; NIDDM: noninsulin-dependent diabetes mellitus; *N*: number of subjects; *P*: statistical significance level (*P* < 0.005).

**Table 2:** Pupil diameter (mm).

	Group 1: Drops		Group 2: Mydriasert		
	Mean	SD	Mean	SD	*P*
15 minutes	5.78	1.86	4.64	1.49	0.011
30 minutes	7.83	1.69	7.67	2.44	0.75
45 minutes	8.71	1.68	9	1.75	0.564
60 minutes (presurg)	9.44	1.17	9.05	1.54	0.481
End of surgery	6.73	1.51	6.72	0.82	0.99
24 hours postop	5.20	1.54	3.33	1.15	<0.001

*N*	35		35		

SD: standard deviation; presurg: presurgical; postop: postoperative; *N*: number of subjects; *P*: statistical significance level (*P* < 0.005).

**Table 3:** Blood pressure and heart rate in both groups during the study.

	Group 1: Drops		Group 2: Mydriasert		
	Mean	SD	Mean	SD	*P*
HR 15 min. (bpm)	66.77	10.09	71.21	10.89	0.099
HR 30 min. (bpm)	65.83	10.34	68.44	11.09	0.34
HR 45 min. (bpm)	66.70	10.0	71.0	10.95	0.18
HR postop (bpm)	66.81	9.94	70.55	11.52	0.23
SBP 15 min.	133.69	26.5	134.81	25.7	0.86
SBP 30 min.	126.34	22.97	132.11	25.10	0.35
SBP 45 min.	125.48	16.93	127.80	17.95	0.66
SBP postop	138.26	28.02	143.55	24.16	0.48
DBP 15 min.	69.66	13.78	71.07	10.88	0.66
DBP 30 min.	67.89	11.59	69.44	10.09	0.58
DBP 45 min.	67.65	10.59	70.50	11.58	0.40
DBP postop	66.85	11.10	72.55	8.19	0.60

*N*	35		35		

HR: heart rate; bpm: beats per minute; postop: postoperative; SBP: systolic blood pressure; postop: postoperative; DBP: diastolic blood pressure; *N*: number of subjects; SD: standard deviation; *P*: statistical significance level (*P* < 0.005).
